# Adjusting for the diagnostic accuracy of CXR in the dose-response relationship between cumulative silica exposure and silicosis in miners

**DOI:** 10.1136/oemed-2025-110368

**Published:** 2026-07-09

**Authors:** Patrick Howlett, Ashwin Durairaj, Jeffrey Gan, Maia Lesosky, Johanna Feary

**Affiliations:** 1 Imperial College London National Heart and Lung Institute, London, UK; 2 Imperial College London Faculty of Medicine, London, UK

**Keywords:** Silicosis, Materials, exposures or occupational groups, Coal Mining, Lung Diseases, Interstitial, Construction Industry

## Abstract

**Introduction:**

A recent meta-analysis confirmed that chest X-ray (CXR) has low sensitivity for diagnosing silicosis. We re-estimated previously published dose-response relationships between cumulative respirable crystalline silica (RCS) exposure and silicosis risk, under the assumptions that sensitivity was either fixed or relative to the population proportion of severe silicosis.

**Methods:**

We combined unpublished logistic regression models from Scottish coal miners with meta-analysis results to model how CXR sensitivity changed according to cumulative RCS exposure. We assumed specificity was 0.95. Among mining cohorts, we calculated the difference in the cumulative risk of silicosis between the unadjusted and fixed and relative scenarios. Finally, we re-estimated a published dose-response meta-analysis and associated absolute risk reductions (ARR).

**Results:**

The cumulative risk of silicosis was substantially higher in both the fixed and relative sensitivity scenarios compared with the unadjusted estimate in all mining cohorts. This was most pronounced in the relative scenario and when cumulative RCS exposures were below approximately 6 mg/m³-years. A reduction in cumulative RCS exposure from 4 to 2mg/m³-years corresponded to larger ARRs in the fixed and relative scenarios than the unadjusted scenario; 382 (95% CI 361 to 399) and 529 (95% CI 353 to 592) cases per 1000 miners compared with 313 (95% CI 288 to 333) cases per 1000 miners, respectively.

**Discussion:**

We relied on a single estimate of the proportion of severe disease to link sensitivity and cumulative RCS exposure. Nevertheless, adjusting for the reduced diagnostic accuracy of CXR for silicosis suggests the burden of silicosis is underestimated in published mining cohorts.

**WHAT IS ALREADY KNOWN ON THIS TOPIC**

- Despite its reduced diagnostic accuracy for silicosis, chest X-ray (CXR) has been used for silicosis diagnosis in all major studies of the dose-response relationship between cumulative silica exposure and silicosis.

**WHAT THIS STUDY ADDS**

- Adjusting for the reduced sensitivity and specificity of CXR for silicosis in previously published studies resulted in meaningful increases in the risk of silicosis at the lower range exposures frequently encountered in the workplace (less than 6 mg/m^3^-years).

**HOW THIS STUDY MIGHT AFFECT RESEARCH, PRACTICE OR POLICY**

- When considering thresholds for silica exposure, policymakers and researchers should interpret existing CXR-based studies of silicosis in the context of a clinically meaningful underestimation of silicosis risk.

## Introduction

 The global resurgence of silicosis has prompted reviews of the dose-response relationship and disease thresholds between silica exposure and silicosis risk.^1–3^ All the reviewed studies use chest X-ray (CXR) as the diagnostic method for silicosis. However, one of the main sources of bias has been the proportion of silicosis present on high-resolution CT (HRCT) or autopsy that is not visible on CXR.^4^

We previously demonstrated the reduced sensitivity of CXR for silicosis, compared with HRCT or autopsy under two counterfactual scenarios.^5^ First, assuming sensitivity of CXR was fixed, we estimated sensitivity to be 0.76 (95% CI 0.63 to 0.86) compared with the HRCT. Second, assuming that CXR sensitivity was explained by the proportion of severe silicosis in the population, we estimated that sensitivity increased by 1.15% (95% CI 0.44% to 1.86%) for each 1% increase in the proportion of severe silicosis. Severe silicosis was defined as the proportion of International Labour Organization (ILO) classification category ≥2/1 relative to ≥1/0.^5^ Specificity (0.89, 95% CI 0.77 to 0.95) was likely falsely low due to selection bias.

As silicosis risk is related to cumulative silica exposure, by extension, the sensitivity of CXR for silicosis may be related to cumulative silica exposure. Again, the relationship may be fixed or, as previously suggested by Hnizdo *et al*,^6^ relative. Given the potential magnitude of the error in silicosis case numbers and as current policy documents reference risk estimates from these studies, adjusting for the reduced sensitivity and specificity of CXR for silicosis is important.^1^

In this analysis, we modelled how CXR sensitivity changed according to cumulative silica exposure. Under fixed and relative sensitivity and fixed specificity scenarios, we re-estimated cumulative risk and the dose-response relationship between cumulative silica exposure and silicosis among miner cohorts.

## Methods

The fixed sensitivity scenario assumed CXR sensitivity to be 0.76 (95% CI 0.63 to 0.86).^5^ To estimate relative sensitivity, we obtained unpublished logistic regression models of silicosis risk from a study of Scottish coal miners^7 8^ and calculated the proportion of CXR ILO category ≥2/1 silicosis relative to ≥1/0 according to cumulative respirable crystalline silica (RCS). Using these values in our previously published meta-regression, we estimated the corresponding (relative) sensitivity of CXR according to cumulative RCS exposure. Example calculations are provided in online supplemental table 2. We heuristically chose 0.95 (the upper CI limit in our meta-analysis) as a fixed specificity value.

We re-estimated the true positive, true negative, false positive and false negative values of silicosis in each category of cumulative RCS exposure of miner cohorts in our previous review.^1^ We estimated cumulative risk using the Steenland method.^9^ As previously, we fitted non-parametric locally weighted least squares estimates (LOESS) and extracted median and quartile cumulative risks at exposures of 1, 2, 4 and 10 mg/m^3^-years. For fixed and relative sensitivity scenarios, using previous methods,^1^ we recalculated the dose-response meta-analysis and absolute risk reduction (ARR) compared with a baseline of 4 mg/m^3^-years cumulative exposure (equivalent to 40 years at 0.1 mg/m^3^). To reflect our range of greatest clinical interest, a secondary analysis used empirical knot values of 1, 2, 4, 6 mg/m^3^-years. A sensitivity analysis assumed perfect specificity (1.00). We made two assumptions. For the cumulative risk analysis, the maximum number of cases could not exceed the total number in the category. For the dose-response analysis, as non-infinite risk ratios are required, the maximum possible number was the total number of cases, minus one. We created a web-based application that allows adjustment of sensitivity and specificity and calculates the observed and adjusted risks of the cohorts (https://phowlett.shinyapps.io/silicosis-risk-app/).

Code and data are available at https://github.com/pjhowlett/da_silic_cxr/tree/main.

## Results

Under the relative sensitivity scenario, CXR sensitivity increased from 0.29 when cumulative silica exposure was zero to 1 at 10.14 mg/m³-years (online supplemental figure 1 and table 2).

**Figure 1:**
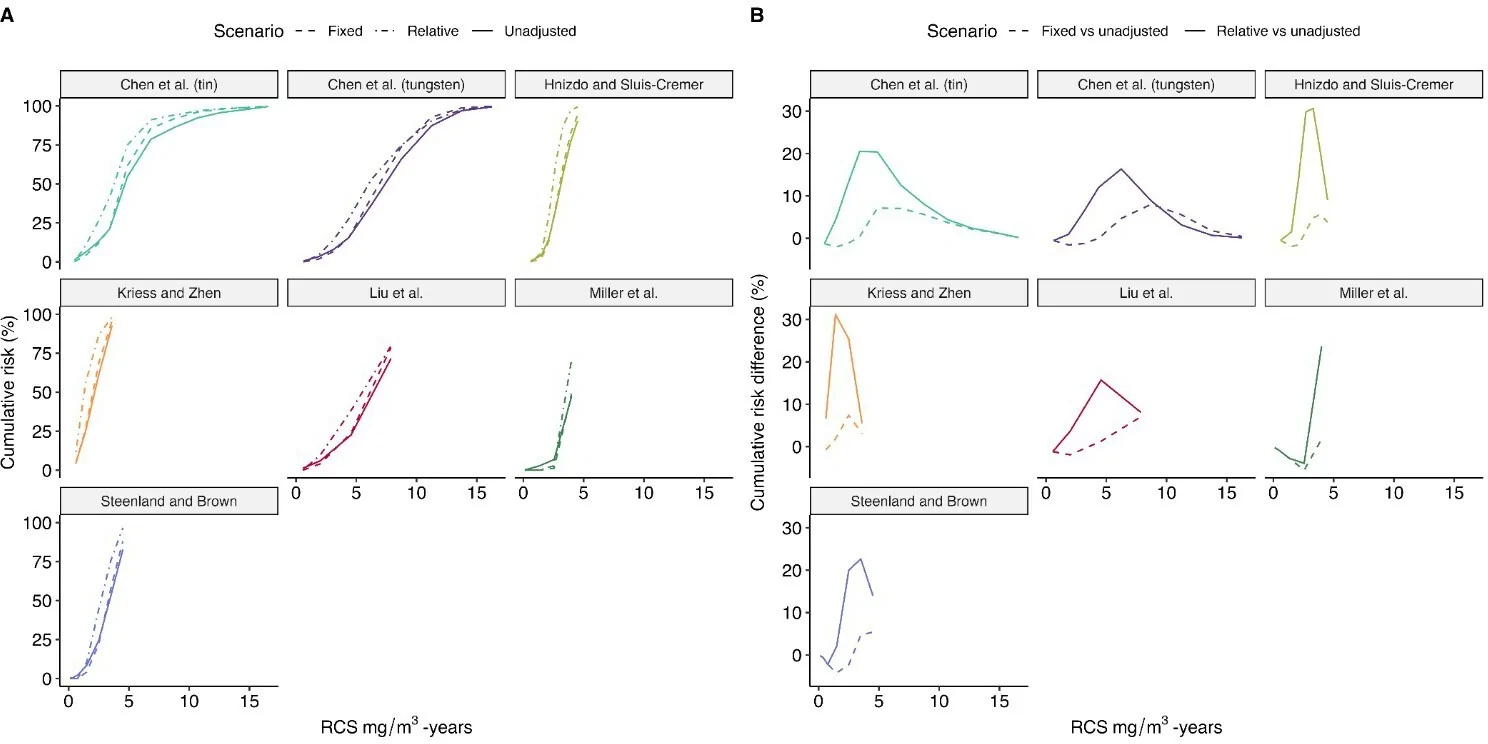
Comparison of cumulative risk of silicosis among studied cohorts of miners (gold, metals, hard rock and coal—details in online supplemental table 1) providing categorical data (n=6) as reported by the study (unadjusted; solid line) and modelled under an assumption of a fixed sensitivity of CXR compared with HRCT (fixed; dashed line) and a relative sensitivity of CXR compared with HRCT (relative; dot-dash line). Cumulative risks were calculated using the Steenland method. Values differ very marginally from previously published estimates^1^; to avoid negative risk estimates, we reduced the LOESS span from 0.75 to 0.5. The studies are represented by the same positioning and colour in plots A and B. (**A**) Comparison of cumulative risks according to cumulative silica exposure for eight cohorts across six studies. The solid line represents the unadjusted risk, dashed the fixed risk and dot-dash the relative risk. (**B**) Representation of the data as the difference between unadjusted risk and adjusted scenarios of fixed and relative sensitivity, according to cumulative silica exposure. The solid line represents the difference between the fixed versus unadjusted risk, the dashed line represents the difference between the relative and unadjusted risk. CXR, chest X-ray; HRCT, high-resolution CT; LOESS, locally weighted least squares estimates; RCS, respirable crystalline silica.

The cumulative risk of silicosis was higher in the fixed and relative sensitivity scenarios compared with the unadjusted estimate in most settings (figure 1). It was most pronounced in the relative sensitivity scenario when exposures ranged between approximately 2 and 6 mg/m^3^-years. However, in the fixed sensitivity scenario, at exposures of approximately 3 mg/m^3^-years and under the cumulative risk of silicosis was lower than the unadjusted value. This is due to false positive results (sometimes all observed cases) which occurred in both the relative and fixed scenarios at lower exposure levels (online supplemental table 3). As the cumulative risks of the studies approached 100%, the differences between scenarios reduced. The median cumulative risk at 4 mg/m^3^-years for the unadjusted, fixed and relative scenarios was 41% (IQR 24%, 61%), 43% (IQR: 25%, 66%) and 63% (IQR 40%, 81%), respectively (online supplemental table 4). Different sensitivity and specificity thresholds may be visualised in our web application (https://phowlett.shinyapps.io/silicosis-risk-app/).

At lower cumulative RCS ranges (approximately <5 mg/m^3^-years) the dose-response curves for the three scenarios were similar (online supplemental figure 2 and 3) following which they diverged. Compared with the primary analysis, empirical knot choices demonstrated a similar pattern at the lower exposure ranges (online supplemental figure 4) but a different pattern at higher ranges. In all analyses, CIs were wide above 5 mg/m^3^-years.

When the absolute risk of silicosis at 4 mg/m^3^-years was fixed at the LOESS-derived median cumulative risk estimate, the ARR in silicosis cases, when cumulative exposure was reduced to 2 mg/m^3^-years, in unadjusted, fixed and relative scenarios was 313 (95% CI 288 to 333) per 1000 miners, 382 (95% CI 361 to 399) per 1000 miners and 529 (95% CI 353 to 592) per 1000 miners, respectively (further exposure level estimates in online supplemental table 5). Secondary and sensitivity analysis results were similar (online supplemental table 6 and 7).

## Discussion

The reduced diagnostic accuracy of CXR for silicosis has limited our understanding of cumulative silica exposure and silicosis risk.^5^ We demonstrated that—when compared with the unadjusted risk—assuming either a fixed sensitivity for CXR or a sensitivity relative to the proportion of severe silicosis in the population results in substantial increases to the cumulative risk of silicosis among miners. This was most pronounced at lower ranges of cumulative exposure (eg, under 6 mg m^3^-years) which may be encountered more frequently in the workplace.

At lower exposures, when prevalence was correspondingly low, reduced specificity resulted in false positives. Under the assumption of fixed sensitivity this resulted in an overall reduction in cumulative risk. However, our assumption of a fixed specificity of 0.95 may be an underestimate of the true specificity of CXR for silicosis. Importantly, the risk of false positive results suggests that confirmatory HRCT scanning is useful, particularly in lower exposed workers with a positive CXR.

Regardless of the risk of overdiagnosis at low exposures, we estimated that substantially more cases were missed under the fixed and relative scenarios, compared with the unadjusted scenario, when cumulative silica exposure was reduced from 4 to 2 mg/m^3^-years. This finding was robust under secondary and sensitivity analyses. That both plausible, yet counterfactual, assumptions of fixed and relative sensitivity demonstrate substantial differences from the unadjusted estimates lends weight to the conclusion that a true, clinically important underestimation of silicosis risk in these studies exists. This is important because the individual studies we included^1^ have been used by researchers and legislators of exposure limits.^2^ Our results suggest future interpretations should assume silicosis risk was underestimated at most exposure levels in these studies.

In addition to individual study limitations, our methods have limitations. We used the HRCT reference standard for sensitivity which may underestimate silicosis risk compared with an autopsy reference.^5^ To estimate proportions of severe disease, we used coefficients from models of 50–75 years old miners with over 20 years latency and exposure data with less than 2 mg/m^3^ intensity.^7^ Although a comprehensive and well-regarded study of silicosis among Scottish coal miners,^2^ our application may not have been generalisable.^10^ Misclassification due to mixed dust pneumoconiosis may have occurred, although this is likely non-differential with respect to the proportion of severe to less severe disease. Furthermore, the assumption that predicted probabilities from a logistic regression equate to population level estimates is simplistic. However, to the best of our knowledge, it is the only available data that provides estimates of ILO category risk according to cumulative exposure. Secondary analysis with different knot locations demonstrated that higher meta-analysis values were sensitive to knot placement, although subsequent ARRs at lower exposures were similar. Importantly, while our findings can be useful, due to multiple necessary assumptions we caution that the results should be viewed as illustrative rather than precise or predictive estimates. Finally, this paper does not recommend or oppose HRCT screening for silicosis (a question which requires further study) but provides a re-interpretation of previous studies which have been, and are likely to continue to be, used for policy and research purposes.

In conclusion, currently reported estimates of silicosis risk according to cumulative silica exposure are likely clinically relevant underestimates due to the low sensitivity of CXR for silicosis.

## Supplementary material

10.1136/oemed-2025-110368 online supplemental file 1

## Data Availability

Data are available on reasonable request. All data relevant to the study are included in the article or uploaded as supplementary information.
