# Supplementary material for: Adjusting for the diagnostic accuracy of CXR in the dose-response relationship between cumulative silica exposure and silicosis in miners
Source: Occup Environ Med. 2026 Jul 9;83(5):e110368. doi: 10.1136/oemed-2025-110368 (PMC13539859; doi:10.1136/oemed-2025-110368)
Supplement: Supplementary file 1 [file oemed-83-5-s001.docx]

**Supplement**

**Adjusting for the reduced sensitivity of CXR in the dose-response relationship between cumulative silica exposure and silicosis in miners**

**Author list and affiliations:**

1. *Patrick Howlett MBChB BSc MSc^1^ – [p.howlett@imperial.ac.uk](mailto:p.howlett@imperial.ac.uk)
2. *Ashwin Durairaj BSc^1^ – [ashwin.durairaj20@imperial.ac.uk](mailto:ashwin.durairaj20@imperial.ac.uk)
3. Jeffery Gan BSc^1^ - [jeffrey.gan19@imperial.ac.uk](mailto:jeffrey.gan19@imperial.ac.uk)
4. Maia Lesosky BSc MSc PhD^1^ – [m.lesosky@imperial.ac.uk](mailto:m.lesosky@imperial.ac.uk)
5. Johanna Feary BM BS MSc FRCP PhD^1,2^ – [j.feary@imperial.ac.uk](mailto:j.feary@imperial.ac.uk)

Table of Contents

[Supplementary table 1.	2](#_Toc218956216)

[Supplementary table 2.	4](#_Toc218956217)

[Supplementary table 3.	5](#_Toc218956218)

[Supplementary table 4.	8](#_Toc218956219)

[Supplementary table 5.	9](#_Toc218956220)

[Supplementary table 6.	10](#_Toc218956221)

[Supplementary table 7.	11](#_Toc218956222)

[Supplementary figure 1.	12](#_Toc218956223)

[Supplementary figure 2.	13](#_Toc218956224)

[Supplementary figure 3	14](#_Toc218956225)

[Supplementary figure 4.	15](#_Toc218956226)

Supplementary table 1. Summary table of included studies with references

| Study (year) | Industry / location | Design & follow-up | Silicosis cases / N (%) | Exposure (mean intensity) | Diagnostic criteria |
| --- | --- | --- | --- | --- | --- |
| Hnizdo & Sluis-Cremer (1993) [1] | Gold mining, South Africa | Cross-sectional with long post-employment follow-up (mean service 24 y) | 313/2235 (14%) | 0.087 mg/m³ | ILO ≥1/1; blinded readers; autopsy validation |
| Steenland & Brown (1995) [2] | Gold mining, USA | Cohort with radiographic surveys and mortality follow-up (avg 37 y) | 170/3330 (5%) | 0.09 mg/m³ | ILO ≥1/1 or small opacities; death certificates |
| Chen et al. (2005) [3] | Tin, tungsten mines; pottery (not included in this brief report), China | Large cohorts with annual radiographs (≥26 y follow-up) | 4456 total (~17–21% by sector) | 0.152–0.254 mg/m³ | Chinese stages ≥I (ILO-equivalent) |
| Liu et al. (2013) [4] | Metal mines; pottery, China | Cohorts with annual radiographs (avg follow-up 34.5 y) | 5297/34,018 (16%) | 0.08–0.52 mg/m³ | Chinese stages ≥I |
| Kreiss & Zhen (1996) [5] | Hard-rock mining, USA | Cross-sectional (mean latency 36 y) | 32/100 (32%) | 0.06–0.08 mg/m³ | ILO >1/0; median of 3 B readers |
| Miller et al. (1998) [6] | Coal mining, Scotland | Survivor cohort with serial radiographs (≥13 y follow-up) | 47/547 (9%) | Not reported | ILO ≥2/1; median of 3 readers |

**Bibliography**

1.	Hnizdo E, Sluis-Cremer GK. Risk of silicosis in a cohort of white South African gold miners. Am J Ind Med [Internet]. 1993 Oct [cited 2022 Oct 25];24(4):447–57. Available from: https://onlinelibrary.wiley.com/doi/10.1002/ajim.4700240409

2.	Steenland K, Brown D. Silicosis among gold miners: exposure--response analyses and risk assessment. Am J Public Health [Internet]. 1995 Oct [cited 2022 Oct 25];85(10):1372–7. Available from: https://ajph.aphapublications.org/doi/full/10.2105/AJPH.85.10.1372

3.	Chen W, Hnizdo E, Chen JQ, Attfield MD, Gao P, Hearl F, et al. Risk of silicosis in cohorts of Chinese tin and tungsten miners, and pottery workers (I): An epidemiological study. Am J Ind Med [Internet]. 2005 July [cited 2023 Feb 7];48(1):1–9. Available from: https://onlinelibrary.wiley.com/doi/10.1002/ajim.20174

4.	Liu Y, Steenland K, Rong Y, Hnizdo E, Huang X, Zhang H, et al. Exposure-Response Analysis and Risk Assessment for Lung Cancer in Relationship to Silica Exposure: A 44-Year Cohort Study of 34,018 Workers. American Journal of Epidemiology [Internet]. 2013 Nov 1 [cited 2022 Oct 25];178(9):1424–33. Available from: https://academic.oup.com/aje/article-lookup/doi/10.1093/aje/kwt139

5.	Kreiss K, Zhen B. Risk of silicosis in a Colorado mining community. Am J Ind Med [Internet]. 1996 Nov [cited 2023 Dec 7];30(5):529–39. Available from: https://onlinelibrary.wiley.com/doi/10.1002/(SICI)1097-0274(199611)30:5<529::AID-AJIM2>3.0.CO;2-O

6.	Miller BG, Hagen S, Love RG, Soutar CA, Cowie HA, Kidd MW, et al. Risks of silicosis in coalworkers exposed to unusual concentrations of respirable quartz. Occupational and Environmental Medicine [Internet]. 1998 Jan 1 [cited 2022 Oct 25];55(1):52–8. Available from: https://oem.bmj.com/lookup/doi/10.1136/oem.55.1.52

Supplementary table 2. Example table of calculation of sensitivity, according to silica exposure, using the Scottish Coal miners logistic regression coefficients and our meta-regression formula. Silica exposures were converted from g/hr/m^3^ to mg/m^3^-years with the standard assumption that 1 year is 8 hours work a day for 270 days.

| Cumulative silica exposure (mg/m3-years) | Cumulative silica exposure (g/hr/m3 ) | Probability of ILO >= 1 disease (based on IOM formula) | Probability of ILO >= 2 disease (based on IOM formula) | Ratio of probabilities of >= ILO2 />= ILO 1 | Sensitivity (based on meta-regression formula) |
| --- | --- | --- | --- | --- | --- |
| 1 | 2.16 | 0.16 (0.09, 0.27) | 0.02 (0.01, 0.07) | 0.13 (0.06, 0.27) | 0.31 (0.23, 0.47) |
| 2 | 4.32 | 0.23 (0.12, 0.4) | 0.04 (0.01, 0.15) | 0.16 (0.07, 0.37) | 0.34 (0.24, 0.59) |
| 4 | 8.64 | 0.41 (0.19, 0.68) | 0.1 (0.01, 0.47) | 0.24 (0.07, 0.69) | 0.44 (0.25, 0.95) |
| 6 | 12.96 | 0.63 (0.29, 0.88) | 0.25 (0.02, 0.82) | 0.4 (0.09, 0.93) | 0.62 (0.26, 1) |
| 8 | 17.28 | 0.8 (0.42, 0.96) | 0.5 (0.04, 0.96) | 0.63 (0.11, 1) | 0.88 (0.28, 1) |
| 10 | 21.6 | 0.91 (0.55, 0.99) | 0.75 (0.08, 0.99) | 0.83 (0.14, 1) | 1 (0.32, 1) |

Supplementary table 3. Table of observed and estimated true silicosis cases (calculated using the Rogan-Gladen estimator) with appropriate true positive (TP), true negative (TN), false positive (FP) and false negative (FN) values among A. Mining cohorts and B. Non-mining cohorts. Specificity in all cases = 0.95

A. Mining cohorts

| Study cohort | Scenario | RCS dose (mg/m^3^-yrs) | n | Observed cases | Sensitivity | True cases | TP | FN | TN | FP |
| --- | --- | --- | --- | --- | --- | --- | --- | --- | --- | --- |
| Chen et al. (tin) | Fixed | 0.4825 | 1150 | 37 | 0.76 | 0 | 0 | 0 | 1092 | 37 |
| Chen et al. (tin) | Fixed | 1.4575 | 838 | 103 | 0.76 | 86 | 65 | 21 | 714 | 38 |
| Chen et al. (tin) | Fixed | 2.4325 | 374 | 89 | 0.76 | 99 | 75 | 24 | 261 | 14 |
| Chen et al. (tin) | Fixed | 3.4075 | 307 | 97 | 0.76 | 115 | 87 | 28 | 182 | 10 |
| Chen et al. (tin) | Fixed | 4.87 | 474 | 309 | 0.76 | 402 | 305 | 96 | 69 | 4 |
| Chen et al. (tin) | Fixed | 6.82 | 233 | 157 | 0.76 | 205 | 156 | 49 | 27 | 1 |
| Chen et al. (tin) | Fixed | 8.77 | 53 | 29 | 0.76 | 37 | 28 | 9 | 15 | 1 |
| Chen et al. (tin) | Fixed | 10.72 | 21 | 17 | 0.76 | 21 | 17 | 5 | 0 | 0 |
| Chen et al. (tin) | Fixed | 12.67 | 9 | 8 | 0.76 | 9 | 8 | 2 | 0 | 0 |
| Chen et al. (tin) | Fixed | 14.62 | 4 | 4 | 0.76 | 4 | 4 | 1 | 0 | 0 |
| Chen et al. (tin) | Fixed | 16.575 | 5 | 5 | 0.76 | 5 | 5 | 1 | 0 | 0 |
| Chen et al. (tin) | Relative | 0.4825 | 1150 | 37 | 0.3 | 0 | 0 | 0 | 1092 | 37 |
| Chen et al. (tin) | Relative | 1.4575 | 838 | 103 | 0.32 | 224 | 72 | 152 | 583 | 31 |
| Chen et al. (tin) | Relative | 2.4325 | 374 | 89 | 0.36 | 229 | 82 | 148 | 137 | 7 |
| Chen et al. (tin) | Relative | 3.4075 | 307 | 97 | 0.4 | 232 | 93 | 138 | 72 | 4 |
| Chen et al. (tin) | Relative | 4.87 | 474 | 309 | 0.51 | 474 | 309 | 234 | 0 | 0 |
| Chen et al. (tin) | Relative | 6.82 | 233 | 157 | 0.72 | 217 | 156 | 60 | 16 | 1 |
| Chen et al. (tin) | Relative | 8.77 | 53 | 29 | 0.98 | 28 | 28 | 1 | 23 | 1 |
| Chen et al. (tin) | Relative | 10.72 | 21 | 17 | 1 | 17 | 17 | 0 | 4 | 0 |
| Chen et al. (tin) | Relative | 12.67 | 9 | 8 | 1 | 8 | 8 | 0 | 1 | 0 |
| Chen et al. (tin) | Relative | 14.62 | 4 | 4 | 1 | 4 | 4 | 0 | 0 | 0 |
| Chen et al. (tin) | Relative | 16.575 | 5 | 5 | 1 | 5 | 5 | 0 | 0 | 0 |
| Chen et al. (tungsten) | Fixed | 0.62 | 4061 | 53 | 0.76 | 0 | 0 | 0 | 3858 | 53 |
| Chen et al. (tungsten) | Fixed | 1.87 | 2065 | 185 | 0.76 | 115 | 88 | 28 | 1852 | 97 |
| Chen et al. (tungsten) | Fixed | 3.12 | 1235 | 244 | 0.76 | 257 | 195 | 62 | 929 | 49 |
| Chen et al. (tungsten) | Fixed | 4.37 | 1146 | 320 | 0.76 | 370 | 281 | 89 | 737 | 39 |
| Chen et al. (tungsten) | Fixed | 6.245 | 1388 | 708 | 0.76 | 899 | 684 | 216 | 464 | 24 |
| Chen et al. (tungsten) | Fixed | 8.745 | 1105 | 773 | 0.76 | 1011 | 768 | 243 | 89 | 5 |
| Chen et al. (tungsten) | Fixed | 11.245 | 537 | 411 | 0.76 | 537 | 411 | 129 | 0 | 0 |
| Chen et al. (tungsten) | Fixed | 13.745 | 139 | 114 | 0.76 | 139 | 114 | 33 | 0 | 0 |
| Chen et al. (tungsten) | Fixed | 16.25 | 10 | 8 | 0.76 | 10 | 8 | 2 | 0 | 0 |
| Chen et al. (tungsten) | Relative | 0.62 | 4061 | 53 | 0.3 | 0 | 0 | 0 | 3858 | 53 |
| Chen et al. (tungsten) | Relative | 1.87 | 2065 | 185 | 0.34 | 286 | 96 | 190 | 1690 | 89 |
| Chen et al. (tungsten) | Relative | 3.12 | 1235 | 244 | 0.39 | 540 | 209 | 331 | 660 | 35 |
| Chen et al. (tungsten) | Relative | 4.37 | 1146 | 320 | 0.46 | 633 | 294 | 339 | 487 | 26 |
| Chen et al. (tungsten) | Relative | 6.245 | 1388 | 708 | 0.65 | 1066 | 692 | 375 | 305 | 16 |
| Chen et al. (tungsten) | Relative | 8.745 | 1105 | 773 | 0.98 | 774 | 756 | 18 | 314 | 17 |
| Chen et al. (tungsten) | Relative | 11.245 | 537 | 411 | 1 | 404 | 404 | 0 | 126 | 7 |
| Chen et al. (tungsten) | Relative | 13.745 | 139 | 114 | 1 | 113 | 113 | 0 | 25 | 1 |
| Chen et al. (tungsten) | Relative | 16.25 | 10 | 8 | 1 | 8 | 8 | 0 | 2 | 0 |
| Hnizdo and Sluis-Cremer | Fixed | 0.6 | 741 | 9 | 0.76 | 0 | 0 | 0 | 704 | 9 |
| Hnizdo and Sluis-Cremer | Fixed | 1.5 | 556 | 48 | 0.76 | 28 | 22 | 7 | 501 | 26 |
| Hnizdo and Sluis-Cremer | Fixed | 2.1 | 469 | 85 | 0.76 | 87 | 66 | 21 | 363 | 19 |
| Hnizdo and Sluis-Cremer | Fixed | 2.7 | 318 | 93 | 0.76 | 109 | 83 | 26 | 199 | 10 |
| Hnizdo and Sluis-Cremer | Fixed | 3.3 | 142 | 53 | 0.76 | 65 | 49 | 16 | 73 | 4 |
| Hnizdo and Sluis-Cremer | Fixed | 3.9 | 44 | 20 | 0.76 | 25 | 19 | 6 | 18 | 1 |
| Hnizdo and Sluis-Cremer | Fixed | 4.5 | 11 | 5 | 0.76 | 6 | 5 | 2 | 4 | 0 |
| Hnizdo and Sluis-Cremer | Relative | 0.6 | 741 | 9 | 0.3 | 0 | 0 | 0 | 704 | 9 |
| Hnizdo and Sluis-Cremer | Relative | 1.5 | 556 | 48 | 0.32 | 74 | 24 | 50 | 458 | 24 |
| Hnizdo and Sluis-Cremer | Relative | 2.1 | 469 | 85 | 0.34 | 210 | 72 | 138 | 246 | 13 |
| Hnizdo and Sluis-Cremer | Relative | 2.7 | 318 | 93 | 0.37 | 243 | 89 | 153 | 72 | 4 |
| Hnizdo and Sluis-Cremer | Relative | 3.3 | 142 | 53 | 0.4 | 132 | 53 | 80 | 9 | 0 |
| Hnizdo and Sluis-Cremer | Relative | 3.9 | 44 | 20 | 0.43 | 44 | 20 | 25 | 0 | 0 |
| Hnizdo and Sluis-Cremer | Relative | 4.5 | 11 | 5 | 0.47 | 10 | 5 | 5 | 1 | 0 |
| Kreiss and Zhen | Fixed | 0.6 | 32 | 4 | 0.76 | 3 | 3 | 1 | 27 | 1 |
| Kreiss and Zhen | Fixed | 1.4 | 38 | 10 | 0.76 | 11 | 9 | 3 | 25 | 1 |
| Kreiss and Zhen | Fixed | 2.5 | 18 | 10 | 0.76 | 13 | 10 | 3 | 5 | 0 |
| Kreiss and Zhen | Fixed | 3.6 | 6 | 5 | 0.76 | 6 | 5 | 1 | 0 | 0 |
| Kreiss and Zhen | Relative | 0.6 | 32 | 4 | 0.3 | 10 | 3 | 7 | 21 | 1 |
| Kreiss and Zhen | Relative | 1.4 | 38 | 10 | 0.32 | 30 | 10 | 20 | 8 | 0 |
| Kreiss and Zhen | Relative | 2.5 | 18 | 10 | 0.36 | 18 | 10 | 12 | 0 | 0 |
| Kreiss and Zhen | Relative | 3.6 | 6 | 5 | 0.41 | 6 | 5 | 4 | 0 | 0 |
| Liu et al. | Fixed | 0.565 | 7687 | 236 | 0.76 | 0 | 0 | 0 | 7303 | 236 |
| Liu et al. | Fixed | 2.015 | 5420 | 663 | 0.76 | 552 | 420 | 133 | 4624 | 243 |
| Liu et al. | Fixed | 4.565 | 4866 | 1566 | 0.76 | 1863 | 1416 | 447 | 2853 | 150 |
| Liu et al. | Fixed | 7.875 | 5655 | 2832 | 0.76 | 3590 | 2729 | 862 | 1961 | 103 |
| Liu et al. | Relative | 0.565 | 7687 | 236 | 0.3 | 0 | 0 | 0 | 7303 | 236 |
| Liu et al. | Relative | 2.015 | 5420 | 663 | 0.34 | 1349 | 459 | 889 | 3868 | 204 |
| Liu et al. | Relative | 4.565 | 4866 | 1566 | 0.48 | 3076 | 1476 | 1599 | 1701 | 90 |
| Liu et al. | Relative | 7.875 | 5655 | 2832 | 0.86 | 3135 | 2706 | 429 | 2394 | 126 |
| Miller et al. | Fixed | 0.107 | 109 | 1 | 0.76 | 0 | 0 | 0 | 104 | 1 |
| Miller et al. | Fixed | 0.526 | 110 | 3 | 0.76 | 0 | 0 | 0 | 104 | 3 |
| Miller et al. | Fixed | 1.334 | 109 | 5 | 0.76 | 0 | 0 | 0 | 104 | 5 |
| Miller et al. | Fixed | 2.552 | 110 | 7 | 0.76 | 2 | 2 | 1 | 102 | 5 |
| Miller et al. | Fixed | 4 | 109 | 31 | 0.76 | 36 | 27 | 9 | 69 | 4 |
| Miller et al. | Relative | 0.107 | 109 | 1 | 0.29 | 0 | 0 | 0 | 104 | 1 |
| Miller et al. | Relative | 0.526 | 110 | 3 | 0.3 | 0 | 0 | 0 | 104 | 3 |
| Miller et al. | Relative | 1.334 | 109 | 5 | 0.32 | 0 | 0 | 0 | 104 | 5 |
| Miller et al. | Relative | 2.552 | 110 | 7 | 0.36 | 5 | 2 | 3 | 100 | 5 |
| Miller et al. | Relative | 4 | 109 | 31 | 0.44 | 66 | 29 | 37 | 41 | 2 |
| Steenland and Brown | Fixed | 0.1 | 1530 | 5 | 0.76 | 0 | 0 | 0 | 1454 | 5 |
| Steenland and Brown | Fixed | 0.35 | 740 | 5 | 0.76 | 0 | 0 | 0 | 703 | 5 |
| Steenland and Brown | Fixed | 0.75 | 376 | 15 | 0.76 | 0 | 0 | 0 | 357 | 15 |
| Steenland and Brown | Fixed | 1.5 | 353 | 33 | 0.76 | 22 | 16 | 5 | 315 | 17 |
| Steenland and Brown | Fixed | 2.5 | 206 | 44 | 0.76 | 47 | 36 | 11 | 151 | 8 |
| Steenland and Brown | Fixed | 3.5 | 73 | 42 | 0.76 | 54 | 41 | 13 | 18 | 1 |
| Steenland and Brown | Fixed | 4.5 | 52 | 26 | 0.76 | 33 | 25 | 8 | 18 | 1 |
| Steenland and Brown | Relative | 0.1 | 1530 | 5 | 0.29 | 0 | 0 | 0 | 1454 | 5 |
| Steenland and Brown | Relative | 0.35 | 740 | 5 | 0.29 | 0 | 0 | 0 | 703 | 5 |
| Steenland and Brown | Relative | 0.75 | 376 | 15 | 0.3 | 0 | 0 | 0 | 357 | 15 |
| Steenland and Brown | Relative | 1.5 | 353 | 33 | 0.32 | 56 | 18 | 38 | 282 | 15 |
| Steenland and Brown | Relative | 2.5 | 206 | 44 | 0.36 | 109 | 39 | 70 | 92 | 5 |
| Steenland and Brown | Relative | 3.5 | 73 | 42 | 0.41 | 73 | 42 | 43 | 0 | 0 |
| Steenland and Brown | Relative | 4.5 | 52 | 26 | 0.47 | 52 | 26 | 27 | 0 | 0 |

Supplementary table 4. Table of estimated cumulative risk of silicosis, calculated using the Steenland method, at corresponding cumulative silica exposures. Rows are separated according to unadjusted, fixed sensitivity and adjusted sensitivity scenarios. Summary rows of median and IQR are presented below each section. Presented studies are only mining cohorts. IQR = Interquartile range

|  | **Cumulative risk (%) at corresponding cumulative silica dose** | | | |
| --- | --- | --- | --- | --- |
|  | **1 mg/m^3^-years** | **2 mg/m^3^-years** | **4 mg/m^3^-years** | **10 mg/m^3^-years** |
| Unadjusted scenario | | | | |
| Chen et al. (tin) | 3.8 | 9.7 | 33.9 | 90.4 |
| Chen et al. (tungsten) | 1.2 | 3.7 | 12.8 | 77.9 |
| Hnizdo and Sluis-Cremer | 1.4 | 12.7 | 77.3 |  |
| Kriess and Zhen | 14.9 | 45.5 |  |  |
| Liu et al. | 2.2 | 6.0 | 20.5 |  |
| Miller et al. | 2.1 | 5.1 | 47.3 |  |
| Steenland and Brown | 3.8 | 16.9 | 66.0 |  |
| Median (IQR) | 2.2 (1.8, 3.8) | 9.7 (5.6, 14.8) | 40.6 (23.9, 61.3) | 84.2 (81.0, 87.3) |
| Fixed sensitivity scenario | | | | |
| Chen et al. (tin) | 2.0 | 8.0 | 36.9 | 94.8 |
| Chen et al. (tungsten) | 0.2 | 2.1 | 12.3 | 85.1 |
| Hnizdo and Sluis-Cremer | 0.2 | 10.9 | 82.8 |  |
| Kriess and Zhen | 15.4 | 50.5 |  |  |
| Liu et al. | 0.9 | 4.1 | 21.3 |  |
| Miller et al. | 0.0 | 0.7 | 49.0 |  |
| Steenland and Brown | 0.9 | 13.5 | 71.0 |  |
| Median (IQR) | 0.9 (0.2, 1.5) | 8 (3.1, 12.2) | 43.0 (25.2, 65.5) | 90.0 (87.5, 92.4) |
| Relative sensitivity scenario | | | | |
| Chen et al. (tin) | 5.7 | 18.9 | 55.1 | 96.0 |
| Chen et al. (tungsten) | 0.7 | 5.0 | 23.2 | 83.5 |
| Hnizdo and Sluis-Cremer | 0.9 | 25.2 | 96.9 |  |
| Kriess and Zhen | 33.7 | 73.4 |  |  |
| Liu et al. | 2.1 | 9.7 | 34.7 |  |
| Miller et al. | 0.0 | 1.7 | 70.9 |  |
| Steenland and Brown | 2.3 | 28.3 | 84.9 |  |
| Median (IQR) | 2.1 (0.8, 4) | 18.9 (7.4, 26.8) | 63 (39.8, 81.4) | 89.8 (86.6, 92.9) |

Supplementary table 5. Primary analysis with specificity equal to 0.95. Comparison of the relative risk (as calculated in the dose-response meta-analysis) estimated number of silicosis cases per 1000 miners and absolute risk difference between levels of cumulative silica exposure

| Cumulative silica dose (mg/m^3^-years) | Risk ratio (95% CI) | Estimated numbers of silicosis per 1000 miners (95% CI) | Absolute risk difference compared to cumulative risk at 4 mg/m^3^-years per 1000 persons (95% CI) |
| --- | --- | --- | --- |
| Unadjusted scenario | | | |
| 1 | 0.07 (0.05, 0.1) | 28 (20, 41) | 378 (365, 386) |
| 2 | 0.23 (0.18, 0.29) | 93 (73, 118) | 313 (288, 333) |
| 4 | 1 (1, 1) | 406 (406, 406) | 0 (0, 0) |
| 6 | 2.25 (1.93, 2.62) | 914 (784, 1000) | -508 (-594, -378) |
| Fixed sensitivity scenario | | | |
| 1 | 0.02 (0.01, 0.05) | 9 (4, 21) | 421 (408, 425) |
| 2 | 0.11 (0.07, 0.16) | 47 (30, 69) | 382 (361, 399) |
| 4 | 1 (1, 1) | 430 (430, 430) | 0 (0, 0) |
| 6 | 2.75 (1.92, 3.94) | 1000 (825, 1000) | -570 (-570, -395) |
| Relative sensitivity scenario | | | |
| 1 | 0.02 (0, 0.07) | 13 (0, 44) | 617 (586, 630) |
| 2 | 0.16 (0.06, 0.44) | 101 (38, 277) | 529 (353, 592) |
| 4 | 1 (1, 1) | 630 (630, 630) | 0 (0, 0) |
| 6 | 1.97 (0.42, 9.34) | 1000 (265, 1000) | -370 (-370, 365) |

Supplementary table 6. Secondary analysis with empirical knots and specificity equal to 0.95. Comparison of the relative risk (as calculated in the dose-response meta-analysis) estimated number of silicosis cases per 1000 miners and absolute risk difference between levels of cumulative silica exposure

| Cumulative silica dose (mg/m^3^-years) | Risk ratio (95% CI) | Estimated number of silicosis per 1000 miners (95% CI) | Absolute risk difference compared to cumulative risk at 4 mg/m^3^-years per 1000 persons (95% CI) |
| --- | --- | --- | --- |
| Unadjusted scenario | | | |
| 1 | 0.06 (0.04, 0.09) | 24 (16, 37) | 382 (369, 390) |
| 2 | 0.21 (0.16, 0.27) | 85 (65, 110) | 321 (296, 341) |
| 4 | 1 (1, 1) | 406 (406, 406) | 0 (0, 0) |
| 6 | 1.79 (1.56, 2.06) | 727 (633, 836) | -321 (-430, -227) |
| Fixed sensitivity scenario | | | |
| 1 | 0.02 (0.01, 0.04) | 9 (4, 17) | 421 (412, 425) |
| 2 | 0.09 (0.06, 0.14) | 39 (26, 60) | 391 (369, 404) |
| 4 | 1 (1, 1) | 430 (430, 430) | 0 (0, 0) |
| 6 | 1.92 (1.26, 2.94) | 825 (541, 1000) | -395 (-570, -112) |
| Relative sensitivity scenario | | | |
| 1 | 0.02 (0, 0.07) | 13 (0, 44) | 617 (586, 630) |
| 2 | 0.14 (0.05, 0.44) | 88 (32, 277) | 542 (353, 598) |
| 4 | 1 (1, 1) | 630 (630, 630) | 0 (0, 0) |
| 6 | 1.18 (0.87, 1.6) | 743 (548, 1000) | -113 (-370, 82) |

Supplementary table 7. Primary analysis with the assumption of perfect specificity (specificity = 1.00). Comparison of the relative risk (as calculated in the dose-response meta-analysis) estimated number of silicosis cases per 1000 miners and absolute risk difference between levels of cumulative silica exposure

| Cumulative silica dose (mg/m^3^-years) | Risk ratio (95% CI) | Estimated number of silicosis per 1000 miners (95% CI) | Absolute risk difference compared to cumulative risk at 4 mg/m^3^-years per 1000 persons (95% CI) |
| --- | --- | --- | --- |
| Unadjusted scenario | | | |
| 1 | 0.07 (0.05, 0.1) | 28 (20, 41) | 378 (365, 386) |
| 2 | 0.23 (0.18, 0.29) | 93 (73, 118) | 313 (288, 333) |
| 4 | 1 (1, 1) | 406 (406, 406) | 0 (0, 0) |
| 6 | 2.25 (1.93, 2.62) | 914 (784, 1000) | -508 (-594, -378) |
| Fixed sensitivity scenario | | | |
| 1 | 0.05 (0.03, 0.08) | 21 (13, 34) | 408 (395, 417) |
| 2 | 0.19 (0.14, 0.26) | 82 (60, 112) | 348 (318, 369) |
| 4 | 1 (1, 1) | 430 (430, 430) | 0 (0, 0) |
| 6 | 2.77 (2.28, 3.36) | 1000 (979, 1000) | -570 (-570, -550) |
| Relative sensitivity scenario | | | |
| 1 | 0.05 (0.02, 0.1) | 32 (13, 63) | 598 (567, 617) |
| 2 | 0.21 (0.12, 0.36) | 132 (76, 227) | 498 (403, 554) |
| 4 | 1 (1, 1) | 630 (630, 630) | 0 (0, 0) |
| 6 | 1.56 (1.26, 1.93) | 983 (794, 1000) | -353 (-370, -164) |

Supplementary figure 1. Modelled exposure-response relationship between cumulative silica exposure (mg/m³-years) and the sensitivity of CXR for detecting silicosis. The curve was derived by combining the mixed-effects linear meta-regression model with a logistic regression model from the Institute of Occupational Medicine (IOM) CXR sensitivity increases from a value of 0.29 at an exposure of 0 mg/m³-years to an with increasing silica exposure, reaching 1.0 (100% sensitivity) at 10.14 mg/m³-years. Sensitivity is equal to 0.29 when exposure is zero, because among the few cases estimated among the miners, 11% were of ≥ 2/1 category.

Supplementary figure 2. Log relative risk of silicosis according to cumulative silica exposure among mining cohorts. The colour of the point represents the study. The size of the point is relative to the standard error of the estimate. These are larger in the Fixed and Relative scenario as the number of silicosis in the lowest, reference groups is smaller than the unadjusted scenario. Unadjusted doses are not standardised (doses range 0.1 to 0.9 mg/m^3^-years). Plot A represents the unadjusted scenario (as in Howlett et al, 2024). Plot B represents the fixed sensitivity scenario. Plot C represents the relative sensitivity scenario. Both are adjusted for reduced specificity.

Supplementary figure 3. Dose-response meta-analysis of the relative risk of silicosis according to cumulative respirable crystalline silica (RCS) exposure using a restricted cubic spline model. The reference category with a relative risk of 1 is fixed at 4 mg/m^3^ -years, equivalent to 40 years working at 0.10 mg/m^3^. As the dose-response method would not allow for all persons in a category to achieve the outcome, 4 of the total 77 categories (across 8 cohorts) were combined. Four knots at default quantiles were chosen (0.5, 0.35, 0.65 and 0.95), equivalent to 0.4, 2.0, 3.9 and 12.0 mg/m^3^-years. Plot A represents the unadjusted scenario (as in Howlett et al, 2024). Plot B represents the fixed sensitivity scenario. Plot C represents the relative sensitivity scenario. Both are adjusted for reduced specificity.

Supplementary figure 4. Secondary analysis with empirical knots. Dose-response meta-analysis of the relative risk of silicosis according to cumulative respirable crystalline silica (RCS) exposure using a restricted cubic spline model. The reference category with a relative risk of 1 is fixed at 4 mg/m^3^ -years, equivalent to 40 years working at 0.10 mg/m^3^. As the dose-response method would not allow for all persons in a category to achieve the outcome, 4 of the total 77 categories (across 8 cohorts) were combined. Four knots at empirical quantiles of 1, 2 , 4, 6 mg/m^3^-years were empirically chosen. Plot A represents the unadjusted scenario (as in Howlett et al, 2024). Plot B represents the fixed sensitivity scenario. Plot C represents the relative sensitivity scenario. Both are adjusted for reduced specificity.
